# Cell voltage versus electrode potential range in aqueous supercapacitors

**DOI:** 10.1038/srep09854

**Published:** 2015-04-21

**Authors:** Zengxin Dai, Chuang Peng, Jung Hoon Chae, Kok Chiang Ng, George Z. Chen

**Affiliations:** 1 College of Materials Science and Engineering, Hunan University, Changsha, Hunan, China 410082; 2 Department of Chemical and Environmental Engineering, and Energy and Sustainability Research Division, Faculty of Engineering, University of Nottingham, Nottingham, UK NG7 2RD; 3 Department of Chemical and Environmental Engineering, Faculty of Science and Engineering, University of Nottingham Ningbo China, Ningbo, China 315100

## Abstract

Supercapacitors with aqueous electrolytes and nanostructured composite electrodes are attractive because of their high charging-discharging speed, long cycle life, low environmental impact and wide commercial affordability. However, the energy capacity of aqueous supercapacitors is limited by the electrochemical window of water. In this paper, a recently reported engineering strategy is further developed and demonstrated to correlate the maximum charging voltage of a supercapacitor with the capacitive potential ranges and the capacitance ratio of the two electrodes. Beyond the maximum charging voltage, a supercapacitor may still operate, but at the expense of a reduced cycle life. In addition, it is shown that the supercapacitor performance is strongly affected by the initial and zero charge potentials of the electrodes. Further, the differences are highlighted and elaborated between freshly prepared, aged under open circuit conditions, and cycled electrodes of composites of conducting polymers and carbon nanotubes. The first voltammetric charging-discharging cycle has an electrode conditioning effect to change the electrodes from their initial potentials to the potential of zero voltage, and reduce the irreversibility.

Supercapacitors store electric energy through charge accumulation in the electric double layer, or redox reactions involving delocalised electrons, or a combination of the two^1^,^2^. They have received fast growing interests from academia and industry alike, due to two main merits, high power performance and long cycle life. In comparable terms of these two types of supercapacitors, the specific capacity of energy storage (W_M_ in unit of J g^−1^) is determined by both the specific capacitance (C_M_, F g^−1^) and the cell voltage (U, V) via the simple equation of W_M_ = C_M_ × U^2^ / 2. To achieve high specific capacitance, redox active materials with pseudocapacitance are highly favoured. In the last decade, composites of pseudocapacitive materials and porous or nanostructured carbon with specially engineered functionalities have become a research focus as they combine the high energy and high power of the two components^1^,^2^,^3^. In particular, composites of transition metal oxides or conducting polymers with carbon nanotubes (CNTs) or graphene have exhibited very promising performances^1^,^2^,^3^. In many cases, aqueous electrolytes are used in these supercapacitors with composite electrode materials to take advantages of, such as, low impedance or equivalent series resistance (ESR), low cost, better safety, and low environmental concern^4^,^5^,^6^. However, the main problem of aqueous supercapacitors for commercial applications is the moderate cell voltage as compared to their counterparts with ionic liquid or organic electrolytes^7^,^8^.

Numerous attempts have been made to increase the “maximum charging voltage” (MCV) or the voltage limit of the aqueous supercapacitor^4^,^5^,^9^,^10^,^11^,^12^. One of the common strategies is the selection of electrode materials with wider operational potential ranges. A number of aqueous supercapacitors have demonstrated MCV higher than the thermodynamic breakdown window of water, taking advantage of the stable potential windows of some electrode materials and their high overpotentials for oxygen and/or hydrogen evolution^4^,^5^,^11^,^12^,^13^,^14^,^15^. Most of the reported studies on aqueous supercapacitor voltage focus on electrode materials, particularly activated carbon (AC) of high specific surface areas, or manganese oxides^4^,^5^,^9^,^10^,^11^,^12^,^13^,^14^,^15^. To our best knowledge, very little has been reported in the literature on design and engineering which can potentially offer further cost and performance benefits to the development of supercapacitors.

In this paper, from the viewpoint of device design and engineering, we provide a basic reference of the MCVs of aqueous supercapacitors with a wide range of electrode materials, including composites of CNTs with conducting polymers or transition metal oxides. The other two voltage-related performance indicators, i.e. the coulombic efficiency and cycle life, are also investigated. A special emphasis is given to explain the phenomena of the “potential of zero voltage” (PZV), “capacitive potential range” (CPR) and the “positive to negative electrode capacitance ratio” (C_+_/C_−_), and to use them in a simple model to correlate MCV with CPR of each of the two electrodes in supercapacitors. Finally, we demonstrate a new example to show how the optimal C_+_/C_−_ ratio in the “polypyrrole-CNT (+) | KCl | AC (−)” asymmetrical supercapacitor can extend the MCV, and double the specific energy capacity of the supercapacitor without changing the materials^16^,^17^.

## Results

The aim of this work is to understand electrode behaviour and correlate it with and improve the supercapacitor performance from the point of view of device design and engineering. Therefore, we have undertaken both theoretical and experimental studies. The theoretical studies, as presented in the first two sub-sections below, focus on understanding and clarification of key performance parameters that are unique to supercapacitors, but not to batteries. We then experimentally test the theoretical understanding and predictions on selected known electrode materials using the most commonly used electrochemical method, cyclic voltammetry together with simultaneous monitoring of the electrode potential. For other properties of the selected materials, these have already been reported in the literature and the respective references are given in the relevant discussion below.

### Capacitive potential range (CPR)

Unlike batteries that have a relatively constant working voltage, the voltage of a supercapacitor is proportional to the amount of stored electrical charge, i.e., U = Q / C. The fully charged state of a supercapacitor corresponds to the MCV which is usually determined by any irreversible electrode reaction, such as solvent decomposition and irreversible redox reactions on one of the electrodes. The latter is often referred to as the over-oxidation or over-reduction of the redox active materials. In between the lower and upper potential limits, there is a region where the electrode behaves like a capacitor, e.g. a constant current in cyclic voltammetry, which is defined as the “capacitive potential range” (CPR) of the electrode.

**Figure 1:**
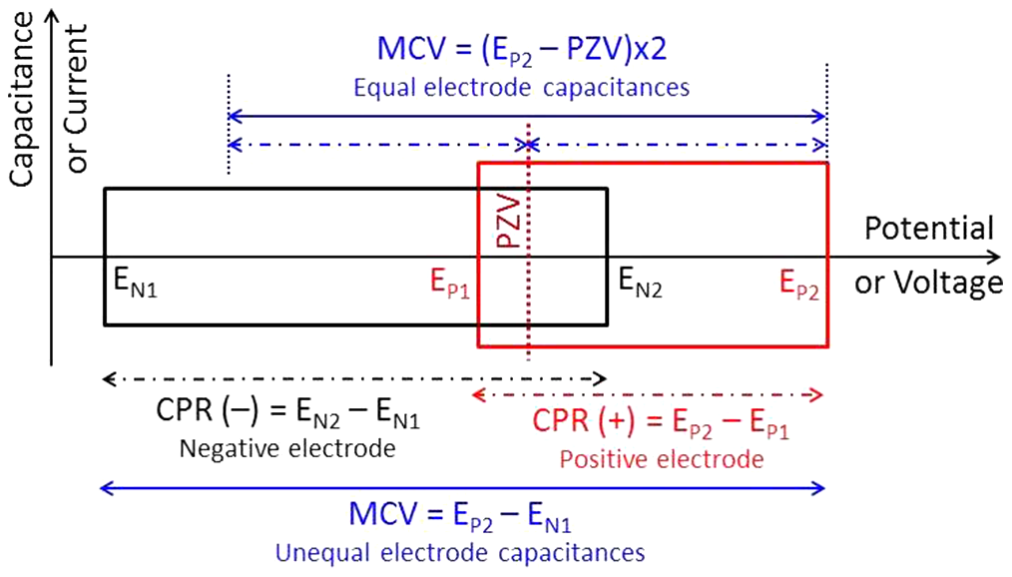
A model of supercapacitor voltage. Schematic illustration of supercapacitor maximum charging voltage (MCV), potential of zero voltage (PZV), and electrode capacitive potential range (CPR: E_N2_ − E_N1_ or E_P2_ − E_P1_ for the negative or positive electrode, respectively).

As shown in Fig. 1, the lower and upper potential limits of the negative and positive electrodes are denoted as E_N1_, E_N2_, E_P1_ and E_P2_, respectively. The CPR of the negative or positive electrode is E_N2_ – E_N1_ or E_P2_ – E_P1_, respectively. In many cases, there is an overlap between the CPRs of the two electrodes, corresponding to E_N2_ > E_P1_, which means the MCV of the supercapacitor is narrower than the sum of the CPRs of the two electrodes. An extreme case is that the CPR of one electrode covers or includes completely that of the other electrode. It can be described as E_N1_ < E_P1_ and E_N2_ > E_P2_ for a wider negative electrode CPR, or E_P1_ < E_N1_ and E_P2_ > E_N2_ for a wider positive electrode CPR. In such cases, the MCV of the supercapacitor is restricted to the narrower CPR. On the other hand, if the CPRs of the two electrodes are not connected, i.e. E_N2_ < E_P1_, the device would then have a “minimum discharging voltage” (MDV = E_P1_ – E_N2_). These extreme cases are of theoretical interest but not necessarily practical or have not yet been practiced. Thus, the following discussion will be on supercapacitors with connected CPRs of the two electrodes, i.e. E_N2_ ≥ E_P1_.

### Potential of zero voltage (PZV)

When a supercapacitor is fully discharged, its voltage is zero if E_N2_ ≥ E_P1_ and the potentials of the two electrodes are equal. This potential is defined as the “potential of zero voltage” (PZV) or equi-potential^17^. The PZV affects the coulombic efficiency and the MCV of a supercapacitor, but is difficult to predict in practice. For an asymmetric supercapacitor, if PZV − E_N1_ > E_P2_ − PZV, and the capacitances of the two electrodes are equal, the MCV would be (E_P2_ − PZV) × 2 (Fig. 1). In this case the positive electrode is the “voltage determining electrode” as the MCV of the supercapacitor is determined by E_P2_. Similarly, if the voltage limit is determined by the lower potential limit of the negative electrode, E_N1_, then PZV − E_N1_ < E_P2_ − PZV, and the voltage limit would become (PZV − E_N1_) × 2.

A previous study has suggested that the MCV of an asymmetrical supercapacitor can be extended by increasing the capacitance of the “voltage determining electrode” relative to that of the other electrode^16^. In such a case, the capacitances of the positive and negative electrodes become unequal, which means a lower specific capacitance of the supercapacitor. However, the increased MCV enables a larger specific energy capacity in the supercapacitor^16^,^17^. Other studies have also found that altering the positive and negative electrode mass ratios can pose an effect on the MCV of a supercapacitor^13^,^18^. Further, this capacitance unequalisation strategy was confirmed to be applicable to supercapacitors with the same material, such as carbon, on both the positive and negative electrodes^17^.

### Cyclic voltammetry of different electrode materials

**Figure 2:**
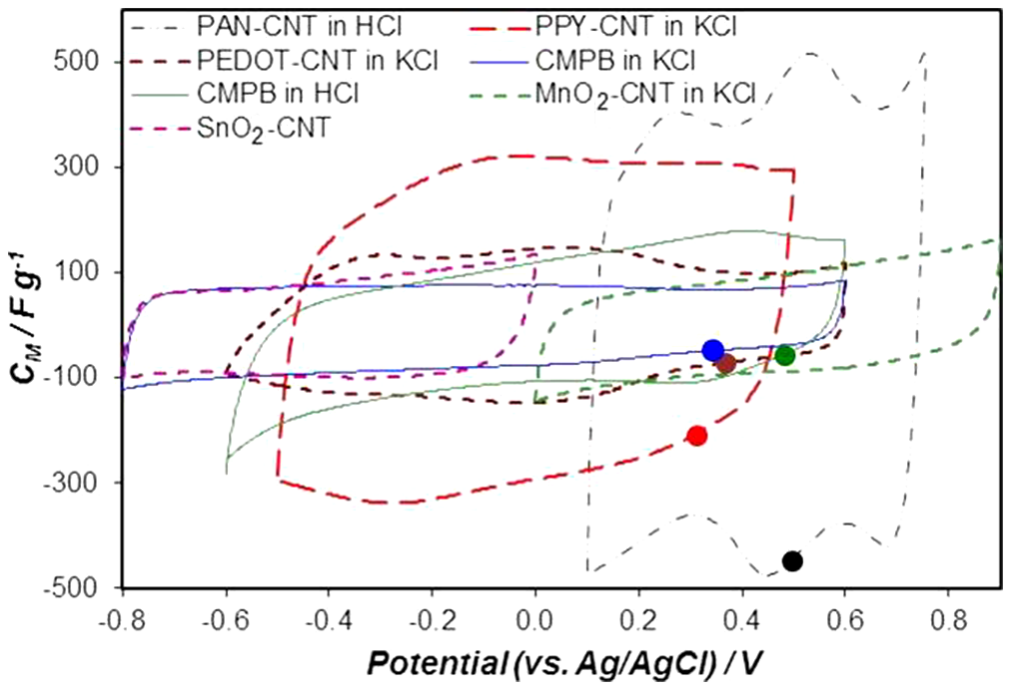
Performances of individual electrodes. Capacitance – electrode potential plots of PAN-CNT, PPY-CNT, PEDOT-CNT, activated carbon (CMPB: Cabot Monarch Pigment Black 1300), MnO_2_-CNT and SnO_2_-CNT. Electrolyte: 0.5 mol L^−1^ KCl or 1 mol L^−1^ HCl as indicated. Reference electrode: Ag/AgCl in 3 mol L^−1^ KCl. The circular marks on some CVs indicate the stable open circuit potentials of the respective electrodes that were measured after immersion in the electrolyte for up to 4 hours.

In the first part of our experimental studies, several known electrode materials were selected and studied for comparison, including the Cabot Monarch 1300 pigment black (CMPB)^6^,^16^,^17^, the various composites of CNTs with polyaniline (PAN-CNT), polypyrrole (PPY-CNT), poly[3,4-ethylene-dioxythiophene] (PEDOT-CNT), manganese dioxide (MnO_2_-CNT) and tin dioxide (SnO_2_-CNT)^19^,^20^. In order to compare the capacitive behaviour and the CPRs of these electrode materials, their cyclic voltammograms (CVs) are normalised to show specific capacitance versus potential, and are superimposed in Fig. 2.

To achieve the same capacitance on the positive and negative electrodes, the amount of electrode material should be inversely proportional to its specific capacitance, i.e., C_P_ × m_P_ = C_N_ × m_N_, where C_P_ and C_N_ are the specific capacitances (F/g), and m_P_ and m_N_ the masses (g) of the positive and negative electrode materials, respectively. The mass of an electrodeposited conducting polymer-CNT composite is proportional to the deposition charge which is therefore reported below as a proportional indicator of the mass loading on the electrode^19^.

**Figure 3:**
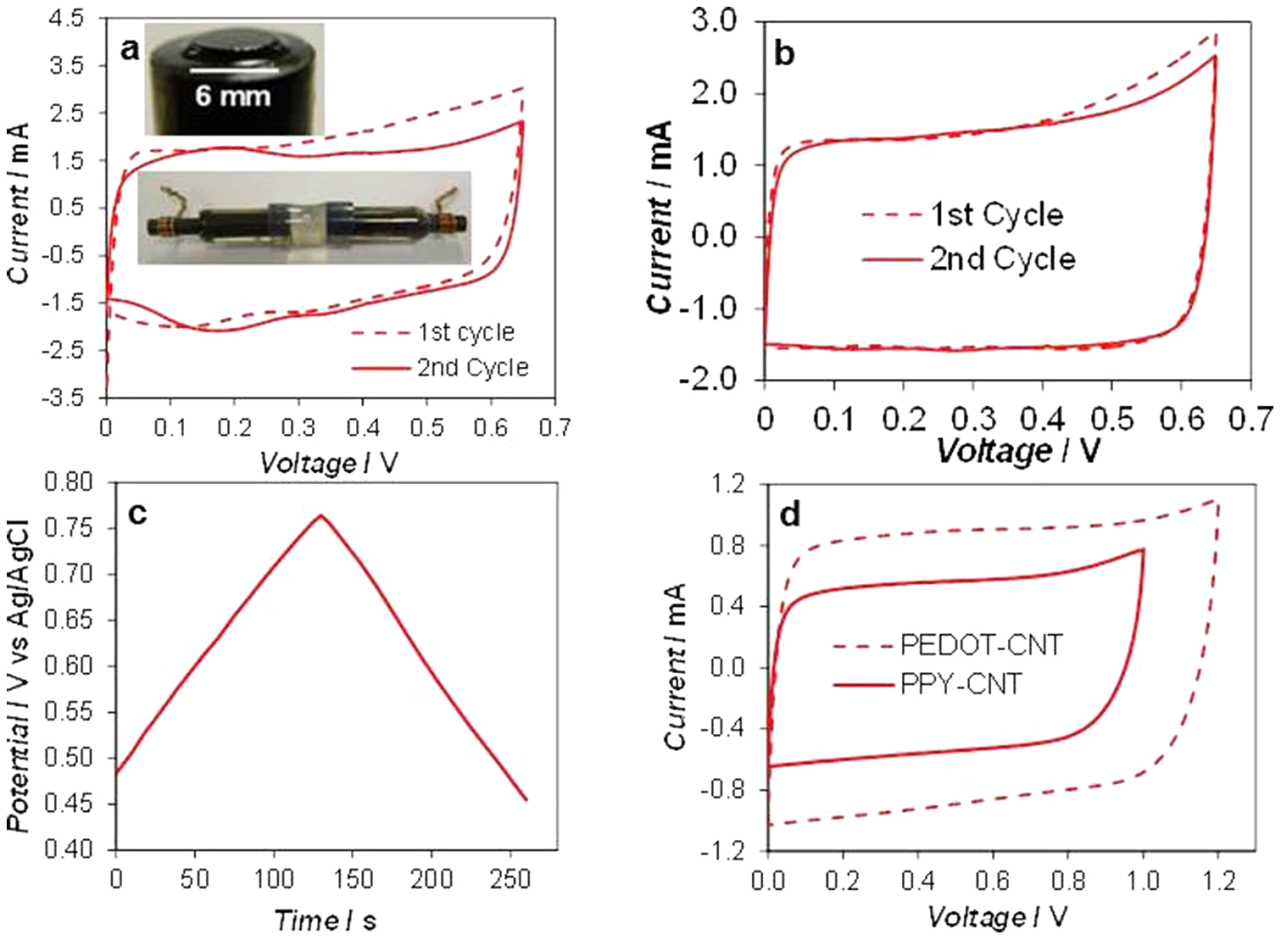
Voltages of symmetrical supercapacitors. Cyclic voltammograms of symmetrical supercapacitors with electrodes of electrodeposited (a,b) PAN-CNT in 1.0 mol L^−1^ HCl (deposition charge: 3.0 C, voltage scan rate: 5 mV s^−1^), (d) PPY-CNT (solid line, 2.8 C, 5 mV s^−1^) and PEDOT-CNT (dashed line, 3.0 C, 10 mV s^−1^) in 0.5 mol L^−1^ KCl. The PAN-CNT electrodes were fresh in (a) and aged under open circuit for 4 hours in (b). (c) Electrode potential of the positive electrode simultaneously recorded with the first cycle CV in (b). Insets in (a): Photographs of the PAN-CNT electrode (showing the electro-deposited PAN-CNT coating on a 6 mm diameter graphite disc) and the tube-cell supercapacitor.

All the CVs in Fig. 2 are approximately rectangular in shape with a current increase at the upper and/or lower potential limits. A rectangular CV corresponds to capacitive behaviour which defines the “CPR” as mentioned above. The current increase at the upper or lower potential limit indicates a non-capacitive electrode processes due to changes in either electrolyte or electrode, or both. Further extending the potential range beyond the limits will possibly result in loss of capacitance during repeated charge-discharge cycles. For CMPB, the positive and negative potential limits are the evolution of oxygen and hydrogen, respectively. Therefore, as shown in Fig. 2, the CMPB electrode works at a less negative potential (−0.6 V) in the acid HCl due to the high proton concentration than in a neutral electrolyte of KCl (−0.8 V). The negative potential limits of the conducting polymers are determined by either the hydrogen evolution reaction or conducting polymers becoming insulating in the undoped state. The positive potential limits of conducting polymers are determined by oxygen evolution or irreversible over-oxidation of the polymer. For instance, PAN is oxidised to pernigraniline at very positive potentials and its capacitance decreases upon potential cycling^16^. The CPR of MnO_2_ is limited by the evolution of oxygen and formation of Mn^2+^ at the positive and negative thresholds, respectively. Of the selected materials, SnO_2_ and its CNT composite are the only ones that exhibit pseudocapacitance in a relatively negative potential range (−0.8 ~ 0 V)^20^, and their CPRs have E_N1_ comparable to that of CMPB. The CPRs of other two materials studied, i.e. PPY-CNT and PEDOT-CNT, fall in the middle range of the potential window explored in Fig. 2. Thus, MnO_2_, PAN and their composites with CNTs are more favourable for the positive electrode in a supercapacitor, while CMPB and SnO_2_ or its CNT composite are more suitable as the negative electrode materials. To further study the MCVs of supercapacitors with different combinations of these electrode materials, the tube-cell supercapacitor was fabricated as shown in the inset of Fig. 3a^17^,^21^.

The open circuit potential (OCP) of an electrode is another important factor affecting the performance of supercapacitors. The stable OCPs of different electrodes were measured, exhibiting the following values: 0.484 V for PAN-CNT, 0.290 V for PPY-CNT, 0.296 V for PEDOT-CNT, 0.365 and 0.466 V for CMPB in KCl and HCl, respectively. These OCP values are also marked in Fig. 2. Interestingly none of the OCP values reside in the middle of their respective CPR. Detailed discussion on the relation between OCP and cell voltage will be provided in the next two sections of this paper.

### Symmetrical supercapacitors

The tube-cell supercapacitors were firstly made symmetrical and consisted of electrodes of electro-deposited conducting polymer-CNT composites. The inset of Fig. 3a presents the photo of an electrode with a thick electrodeposited PAN-CNT coating. CVs of the tube-cell with two identical PAN-CNT electrodes are shown in Fig. 3a for freshly prepared electrodes, and Fig. 3b for electrodes that were aged in electrolyte for about 4 hours under open circuit conditions. The fairly rectangular shape of these CVs suggests good electrode kinetic and mass transport properties for capacitive charge storage. The MCV of a symmetrical supercapacitor is generally not wider than the CPR of each of its two electrodes. This is in agreement between the CPR (0.1 ~ 0.7 V vs. Ag/AgCl) of the PAN-CNT electrode as revealed in Fig. 2, and the MCV (0.65 V) of the symmetrical PAN-CNT supercapacitor shown in both Fig. 3a and 3b. Ideally, the PZV of a symmetrical supercapacitor should be in the middle of the electrode CPR, but this is not always the case as it will be discussed later.

Table 1 compares the MCVs of symmetrical supercapacitors with PAN-CNT, PPY-CNT, PEDOT-CNT, and CMPB, and the literature reported MnO_2_-CNT^22^. These are all in good agreement with their respective electrode CPRs. Since the electrodes in a symmetrical supercapacitor are identical, there is no need to differentiate between positive and negative terminals which brings advantages such as ease of manufacturing and use. However, to achieve higher voltages, the asymmetrical design can be more beneficial.

### Asymmetrical supercapacitors with equal electrode capacitances

**Figure 4:**
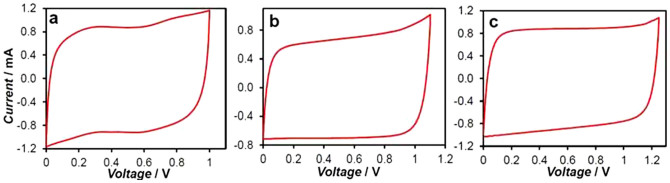
Voltages of asymmetrical supercapacitors. Cyclic voltammograms of asymmetrical supercapacitors of (a) 900 mC PAN-CNT (+) | 1.0 mol L^−1^ HCl | 2 mg CMPB (−), (b) 630 mC PPY-CNT (+) | 0.5 mol L^−1^ KCl | 1 mg CMPB (−), and (c) 3 C PEDOT-CNT (+) | 0.5 mol L^−1^ KCl | 1.7 mg CMPB (−). Voltage scan rates: (a) 10, (b) 20 and (c) 10 mV s^−1^, respectively.

As shown in Fig. 2, the CMPB is a good negative electrode to work together with a pseudocapacitive positive electrode. Thus, asymmetrical tube-cell supercapacitors were constructed with CMPB and conducting polymer-CNT composite as the negative and positive electrode materials respectively. Following the convention, the capacitances of the two electrodes were made equal. Typical results from testing such asymmetrical tube-cells are presented in Fig. 4, confirming the expected larger MCVs. For example, the asymmetrical supercapacitor of “PAN-CNT (+) | HCl | CMPB (−)” had an MCV of 1 V (Fig. 4a), notably higher than that of the PAN-CNT symmetrical capacitor which is about 0.65 V as shown in Fig. 3b.

MnO_2_ has a more positive potential range than the other electrode materials in neutral aqueous electrolytes. Asymmetrical supercapacitors with a MnO_2_ positive electrode have demonstrated high MCVs of 2.0, 1.7, and 1.8 V with activated carbon^5^, SnO_2_-CNT^20^ and PEDOT-CNT^4^ as the negative electrode, respectively. These high MCVs are all feasible considering the well separated CPRs of the selected positive and negative electrodes (Fig. 2). Recent reviews on MnO_2_ also showed high MCVs in asymmetrical supercapacitors with MnO_2_ positive electrodes and neutral aqueous electrolytes^14^,^15^.

Not all the asymmetrical cells listed in Table 1 exhibited an increased MCV. As shown in Fig. 4 and Table 1, the asymmetrical supercapacitor of “PEDOT-CNT (+) | KCl | CMPB (−)” and “PPY-CNT (+) | KCl | CMPB (−)” exhibited only little or no increase in MCV compared to their symmetrical counterparts. More discussions on these cases are given in the following sections.

### Initial electrode potentials and the electrode conditioning process

As listed in Table 1, the MCVs of asymmetrical supercapacitors are generally larger than those of the symmetrical counterparts. These increases can be broadly attributed to the use of a different material for the negative electrode with its CPR being effectively an extension to the negative side of the CPR of the positive electrode. However, it is also obvious that for all the tested supercapacitors, E_N2_ > E_P1_, i.e. there is an overlap between the CPRs of the positive and negative electrodes. In such cases, it is necessary to understand the PZV and its effect on the supercapacitor performance.

It is worth noting that at the PZV, particularly after one or several charging-discharging cycles, the two electrode materials in the supercapacitor are each in a state that is not necessarily the same as that of the material in the freshly constructed supercapacitor. In other words, before any charging-discharging test, the potentials of the two electrodes are highly likely far away from the PZV. For example, the newly electrodeposited PAN-CNT composite is always in the oxidised state, i.e. its open circuit potential is at or close to 0.7 V vs. Ag/AgCl as shown in Fig. 2 (which is E_P2_ of the PAN-CNT composite). When two such electrodes are assembled in the symmetrical tube-cell supercapacitor, the open circuit voltage of the cell could be at or close to 0 V because both electrodes are of the same potential, i.e. 0.7 V where the composite is fully oxidised or charged. This situation differs significantly from that in a fully charged PAN-CNT supercapacitor in which the positive electrode is fully oxidised (0.7 V in Fig. 2) and the negative electrode is fully reduced (0.1 V), or that in a fully discharged supercapacitor in which both the positive and negative electrodes are in the same partially oxidised state (ca. 0.4 V in Fig. 2, which is ideally the PZV). However, the freshly deposited PAN-CNT (and others) is likely in a thermodynamically unstable state (more positive potentials) and may change to a more stable state (less positive potentials) via, for example, relaxation in structure and charge redistribution or interactions with the electrolyte, or both. As a result, if a symmetrical supercapacitor is left for a prolonged period of time, both electrodes will reach more stable OCP values which can be close to or the same as the PZV upon its first charge-discharge cycle. For the PAN-CNT symmetrical supercapacitor in Figure 3b, the OCP of both electrodes after aging under open circuit were measured as 0.483 V. To better understand the change of electrode potential during charging and discharging, the potential of the positive electrode was simultaneously recorded in the course of recording the CVs, and plotted in Figure 3c. Very interestingly, at the end of the first voltage cycle, the electrode potential changed from 0.483 V to 0.455 V. These values were equivalent to the PZV since the cell voltage was zero at the start and end of the voltage cycle.

In practice, the above described situation seems to have, if any, an insignificant impact on supercapacitor research because it has never been mentioned in the literature according to the authors’ best knowledge. In principle, to make a new PAN-CNT symmetrical supercapacitor to work, one of the two PAN-CNT electrodes should be firstly fully discharged or reduced, which requires an oxidation reaction on the other PAN-CNT electrode. Because the other PAN-CNT electrode is already fully oxidised for capacitive charging, further oxidation can only occur via non-capacitive charge transfer, such as over-oxidation of the PAN-CNT and/or oxidation of a component of the electrolyte, e.g. H_2_O.

To test the above analysis and prediction, it is worth revisiting the two consecutive CVs in Fig. 3a which were obtained from the freshly built symmetrical tube-cell supercapacitor of PAN-CNT. At the beginning of the voltage scan (first cycle, near 0 V), a high negative (discharging) current can be seen, which diminished rapidly before the CV became rectangular. This initial large negative current is strong evidence for the above prediction that the negative one of the two new PAN-CTN electrodes must be first reduced (discharged) together with non-capacitive charge transfer on the other. A similar process was actually observed on the first cycle CV of all symmetrical supercapacitors with freshly electrodeposited conducting polymers. When the voltage increased further with the scan, the current passed through zero, and then became positive and more like that for charging the supercapacitor. Note that on the second cycle CV, the unusually large negative current near 0 V seen on the first cycle CV was absent and the CV shape was more rectangular. These differences between the first and second cycle CVs suggest condition changes on both the electrodes. For convenience of discussion, this initial change via both capacitive (discharging) and non-capacitive charge transfer in the supercapacitor is named as “electrode conditioning” which brings the initial potentials of the two electrodes to the PZV in the fully discharged state.

Interestingly, the charging (positive) current of the first cycle CV in Fig. 3a is noticeably larger than that of the second cycle. This can be interpreted by the continuation of the conditioning process at higher voltages. This is because in the first half of the voltage scan (0 to 0.65 V), one of the electrodes (the 1^st^) was reduced but the other electrode (the 2^nd^) remained fully charged, allowing the non-capacitive charge transfer to take place. In the second half of the voltage scan (0.65 to 0 V), however, reduction of the 2^nd^ electrode started although there was no need for non-capacitive oxidisation on the 1^st^ electrode which was already “conditioned”. Calculation showed that the positive and negative charges (Q^+^ and Q^−^) were 1.44 and 1.00 mC during the first voltage cycle, and became 1.17 and 1.05 mC in the second cycle. The coulombic efficiency improved from 69.4% to 89.7%. These differences demonstrate clearly the conditioning effect of the first cycle which must have been responsible for the two electrodes to change to the state of the PZV which should be close to the middle of the CPR of PAN-CNT. It should be pointed out that this conditioning process occurs at the expense of non-capacitive changes to either the electrode or the electrolyte. Fortunately, such non-capacitive changes seem to be chemically reversible or insignificant because the supercapacitors can still perform well in line with the design. For this reason, it would be more prudent to use the second charge-discharge cycle to calculate the capacitance to avoid overestimation.

For the CVs in Fig. 3b, similar features to those in Fig. 3a although less significant, can be seen and attributed to similar causes. In Fig. 3c, the simultaneously recorded PZV of the positive electrode is presented. It can be seen that the PZV changed from 0.483 V to 0.455 V, moving closer towards the middle potential of the CPR of PAN-CNT. As a result, an increase of coulombic efficiency can be expected and was indeed observed. In fact, at the end of the second CV cycle, the PZV further decreased to 0.437 (data not shown), a value that is even closer to the middle potential of the CPR. These results provide direct evidence of the potential conditioning effect during the first or first few charge/discharge cycles.

### Maximum charging voltage (MCV) at unequal electrode capacitances

**Figure 5:**
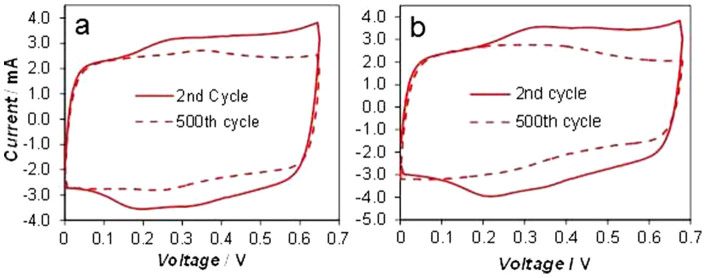
Effect of operating voltage on charge-discharge cycle stability. The 2^nd^ and 500^th^ cyclic voltammograms of PAN-CNT symmetrical supercapacitors with the maximum charging voltage (MCV) of (a) 0.65 V and (b) 0.68 V. Voltage scan rate: 10 mV s^−1^.

The CVs in Figs. 3 and 4 commonly exhibit an increase of current near the high voltage end of the scanned voltage window, indicating some irreversible processes. They provide a reference of the MCV for the construction and safe operation of supercapacitors with different electrode selections. In Fig. 5, the PAN-CNT symmetrical supercapacitor showed initially capacitive CVs at the MCV of 0.65 and 0.68 V. The energy capacity was 9% higher in the latter case. However, after 500 continuous charge-discharge cycles, the current decay was more severe at the MCV of 0.68 V. The capacitance retention was calculated as 81.2% and 77.4% at the MCV of 0.65 and 0.68 V respectively. Thus, there is a trade-off between the MCV and the cycle life of a supercapacitor. This seemingly simple but important fact has rarely been reported in the literature^17^,^22^. It suggests that in order to maximise the cycle life, a supercapacitor should not operate at an inappropriately high voltage.

In relation with the MCV, the supercapacitors mentioned above were fabricated with equal capacitances of the positive and negative electrodes, i.e. C_+_/C_−_ = 1. In such a configuration, the asymmetrical supercapacitors exhibited usually higher MCVs than the symmetrical counterparts. The exceptions are the asymmetrical supercapacitors of “PPY-CNT (+) | KCl | CMPB (−)” and “PEDOT-CNT (+) | KCl | CMPB (−)” whose MCVs were the same as or only slightly larger than those the symmetrical counterpart, “PPY-CNT | KCl | PPY-CNT” and “PEDOT-CNT | KCl | PEDOT-CNT” as shown in Table 1. Further, according to Fig. 2 and Table 1, in some cases, the achieved MCV was still narrower than the sum of the CPRs of the positive and negative electrodes after subtracting the overlapped range. The cause was the equal electrode capacitances as analysed above. It is therefore anticipated that by increasing the relative capacitance of the voltage determining electrode, the MCV of the supercapacitor can further increase. This capacitance unequalisation strategy was previously applied in the asymmetrical supercapacitor of “PAN-CNT (+) | HCl | CMPB (−)”, and even in the aqueous supercapacitor of “CMPB (+) | K_2_SO_4_ | CMPB (−)”^16^,^17^. It was observed that changing the PAN-CNT to CMPB capacitance ratio from 1:1 to 4:3, the MCV increased from 1.05 to 1.40 V. Although raising the capacitance ratio led to a cell specific capacitance loss of about 4.5%, the cell specific energy increased by 73%. Similarly, in the “CMPB (+) | K_2_SO_4_ | CMPB (−)” supercapacitor, the MCV was 1.6 V and 1.9 V at the capacitance ratios of 1:1 and 4:3, respectively. The capacitance loss was 2% but the cell specific energy gain was 38%.

As can be seen in Fig. 2, the CPR of electrodeposited PPY-CNT (−0.5 V to 0.5 V) is enclosed in the CPR of carbon (CMPB, −0.8 V to 0.6 V) in aqueous KCl. Thus, the PPY-CNT electrode should be the voltage determining electrode, and the MCV of the asymmetrical “PPY-CNT (+) | KCl | CMPB (−)” at equal electrode capacitances would not be wider than the CPR of PPY-CNT. To maintain the positive electrode in a positive potential range next to E_P2_ of PPY-CNT, but force the negative electrode into a sufficiently negative potential range near E_N1_ of CMPB, the C_+_/C_−_ ratio must be significantly larger than 1 according to the equation of Q = C_+_ × U_+_ = C_−_ × U_−_, where C_+_ and U_+_ are the capacitance and working potential range of the positive electrode, and C_−_ and U_−_ are those of the negative electrode.

**Figure 6:**
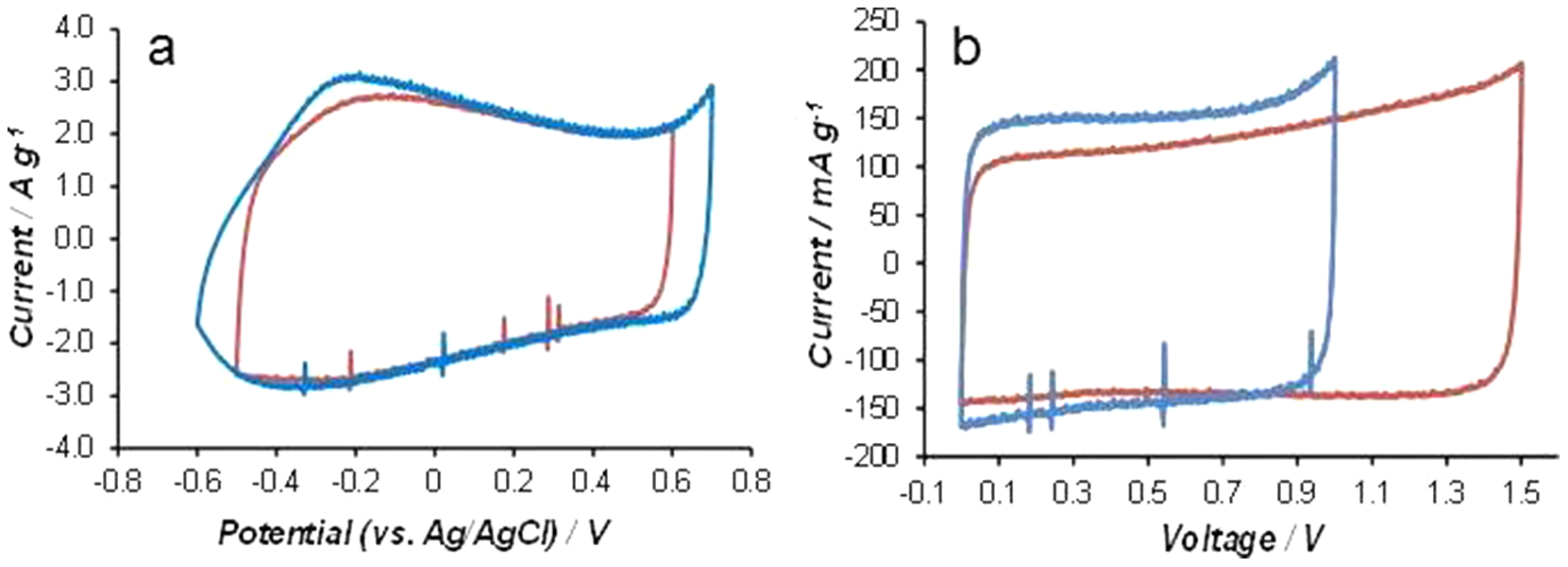
Effect of unequal electrode capacitances on maximum charging voltage. Cyclic voltammograms of (a) chemically synthesised PPY-CNT (20 wt%) in two different potential windows, and (b) asymmetrical supercapacitors of (blue line) “5 mg PPY-CNT (+) | KCl | 10 mg AC (−)” at C_+_/C_−_ = 1.0, and (red line) “24 mg PPY-CNT (+) | KCl | 11 mg AC (−)” at C_+_/C_−_ = 3.0. Specific capacitance: 180 F g^−1^ for PPY−CNT and 125 F g^−1^ for activated carbon. Electrolyte: 3 mol L^−1^ KCl. Scan rate: 5 mV s^−1^.

To further investigate the capacitance unequalisation strategy, a single cell supercapacitor of “PPY-CNT (+) | KCl | AC (−)” was fabricated and studied. In this cell, the AC was a commercial activated carbon (Kuraray, specific surface area: 1500~1800 m^2^ g^−1^). Its specific capacitance was measured to be 125 F g^−1^ in the potential range of −0.9 ~ 0.2 V vs Ag/AgCl in 3 mol L^−1^ KCl. The PPY-CNT used was chemically synthesised as described before^6^ with the CNT content being controlled to be 20 wt.%. The CVs of this chemically synthesised PPY-CNT are presented in Fig. 6a, exhibiting features, particularly the CPR, almost identical to those of the electro-deposited PPY-CNT (Fig. 2).

After a series of tests, it was found that at C_+_/C_−_ = 3.0, the MCV of the “PPY-CNT (+) | KCl | AC (−)” cell was extended to 1.5 V as shown in Fig. 6b. Using a reference electrode and two independent potentiostats^17^, it was observed that the positive and negative electrode potentials were 0.62 V and −0.88 V vs. Ag/AgCl, respectively when the supercapacitor was fully charged to the MCV ( = 1.5 V). The PZV was measured to be 0.17 V when the supercapacitor was discharged to 0 V. In similar measurements of the supercapacitor with equal electrode capacitances, the positive and negative electrode potentials were 0.64 V and −0.36 V vs. Ag/AgCl respectively when the supercapacitor was fully charged to the MCV ( = 1.0 V), whilst the PZV was 0.14 V. By normalisation of the data against the total mass of the two electrodes, it was derived that capacitance unequalisation caused a loss of 9.1% in the cell specific capacitance, but the cell specific energy doubled (29.7 F g^−1^ and 14.9 J g^−1^ at C_+_/C_−_ = 1.0 vs. 27.0 F g^−1^ and 30. 4 J g^−1^ at C_+_/C_−_ = 3.0). It is worth noting that in these two cases, the CPR of the positive PPY-CNT electrode, the PZV, and even E_N2_ of the AC negative electrode remained almost unchanged. The only change was the negative AC electrode CPR becoming significantly wider with E_N1_ shifting from −0.36 to −0.88 V. This finding is unprecedented, but in line with the expected unique role of the “voltage determining electrode” in determining the MCV.

## Discussion

In principle, the performance of a supercapacitor differs, in many ways, from that of other electrochemical devices, particularly rechargeable batteries. However, many concepts and terminologies commonly used for batteries are also used for supercapacitors in the literature. For example, the “potential window” of a battery electrode refers to the potential range in which the changes of the electrode are physically and/or chemically reversible in the charging and discharging processes. However, this concept is not applicable for a supercapacitor electrode in most cases, because in additional to being reversible for charging and discharging, the behaviour of the electrode must be capacitive^2^,^25^. In practical terms, capacitive behaviour refers to a rectangular cyclic voltammogram or a linear potential variation with time in galvanostatic (constant current) charging or discharging.

Surprisingly, in the current literature, we have not found such differentiation and clarification to our satisfaction, and therefore carried out theoretical analyses with an emphasis on device design and engineering. Part of our theoretical findings is reported in this paper in relation with the concepts of “capacitive potential range” and “potential of zero voltage” which are both unique to supercapacitors, but invalid for rechargeable batteries. Another important result from our theoretical studies is the understanding of the effect of electrode capacitance ratio on the energy storage capacity. This understanding was applied to increase the energy capacity of the PAN-CNT | AC and AC | AC supercapacitors^16^,^17^. In this paper, we have reported new findings from optimisation of the electrode capacitance ratio to double the energy capacity of the PPY-CNT | AC supercapacitor. It should be pointed out that in battery designs and fabrication, the two electrodes must be made to have equal charge capacities. Unfortunately, this battery engineering convention has impacted significantly on supercapacitor research and development as evidenced by the concept of “symmetrical supercapacitor” itself. Our studies as reported in this and previous papers^16^,^17^ suggest that it is imperative to design supercapacitors with a careful consideration of the electrode capacitance ratio, disregarding symmetrical or asymmetrical configurations.

In summary, we have studied and compared the maximum charging voltage (MCV) of aqueous supercapacitors with various electrode materials. The capacitive potential ranges (CPRs) and the capacitance ratio of the positive and negative electrodes affect significantly the MCV of a supercapacitor. The potential of zero voltage (PZV) also plays important roles in determining both the MCV and coulombic efficiency of the supercapacitor. The first charge-discharge cycle of a newly built supercapacitor can function as an electrode conditioning process that is effective at the expense of irreversible but insignificant changes to either the electrode and/or electrolyte. However, the conditioning process offers a greater benefit via regulating the PZV and optimising the performances in the following charge-discharge cycles. An asymmetrical supercapacitor of equal electrode capacitances generally has a higher MCV than a symmetrical one if the CPRs of the positive and negative electrodes fall in different but connected potential ranges. If the CPRs overlap significantly, it is necessary to increase the capacitance of the voltage determining electrode (which has a narrow CPR) relative to that of the counter electrode (with a wider CPR) so that the MCV can be further increased. The cycle life of a supercapacitor can be adversely affected when operating at an inappropriately high voltage. A safe cell voltage is vital to ensure high coulombic efficiency and good cycle performances.

## Methods

### Preparation of aqueous suspension of carbon nanotubes

The carbon nanotubes (CNTs, 10–30 nm in diameter, product of chemical vapour decomposition of acetylene, Shenzhen Nanotech Port Co., Ltd.) were heated in concentrated sulfuric and nitric acids under refluxing as described elsewhere^19^. The treatment not only removes impurities such as metal catalyst residues and amorphous carbons, but also introduces carbonyl, carboxyl, and hydroxyl groups to the surface of CNTs, resulting in a stable aqueous suspension^19^. The suspension was then washed and filtered to remove residual acids. The mass percentage of CNTs can reach as high as 1.5%. For the synthesis of MnO_2_-CNT, the suspension was dried at 60°C in an oven and ground with an agate mortar and pestle to produce fine powders of CNTs.

### Electrochemical co-deposition of CNTs and conducting polymers

Pyrrole (99%, Acros Organics), aniline (99.5 +%, Aldrich), and 3,4-ethylenedioxythiophene (EDOT) (98%, Bayer) were used as purchased. The electro-co-deposition of polypyrrole-CNT (PPY-CNT) composite films was carried out in an aqueous solution of 0.25 mol/L pyrrole + 0.3 wt% CNTs. Polyaniline-CNT (PAN-CNT) was prepared from a solution containing 0.25 mol/L aniline + 1.0 mol/L HCl + 0.3 wt% CNTs. Poly[3,4-ethylenedioxythiophene]-CNT (PEDOT-CNT) composite was deposited from a mixture of 5mL acetonitrile and 5 mL water containing 0.25 mol/L EDOT + 0.3 wt% CNTs. All the co-deposition was performed potentiostactically to produce uniform coatings at 0.9, 1.0 and 1.0 V with an Ag/AgCl reference electrode, for PPY-CNT, PAN-CNT and PEDOT-CNT respectively.

### Preparation of MnO_2_–CNT and SnO_2_-CNT by redox deposition and oxidation coating

Suspensions of acid treated CNTs were mixed with neutral aqueous KMnO_4_ solutions of different concentrations to allow oxidation of some surface defect sites of CNTs to CO_3_^2−^ or HCO_3_^−^, leaving a thin coating of MnO_2_ on the surface of individual CNTs. The pH value of the neutral initial solution had increased to 8–9 after the homogenous mixing, an indication of the successful formation of MnO_2_^22^,^23^,^24^. By adjusting the molar ratio of CNTs and KMnO_4_ in the initial solution, the weight ratio of CNTs and MnO_2_ could be controlled^23^,^24^. The composite in this paper had a MnO_2_ : CNT mass ratio of 1:1.

To prepare the SnO_2_-CNT composite, a mixed solution of SnCl_2_ and hydrochloric acid was added to an acid treated CNT suspension. The mixture was stirred for 24 hours at room temperature to allow oxidation of SnCl_2_ by dissolved oxygen and consequently formation of SnO_2_ on the surface of CNTs^20^. The resulting products were filtered, washed, and dried at 60°C.

### Electrode preparation and assembly of tube cell supercapacitor

Composites of CNTs and conducting polymers synthesized by electro-co-deposition form a thin uniform coating on the conducting substrate (Pt or graphite) with good adhesion. However, the nanocomposites synthesized by other methods are all powdery materials. They need to be incorporated with the current collector to form an effective supercapacitor electrode. Chemically polymerized PPY-CNT was mixed with deionised water and 5 wt% polyvinyl alcohol (PVA) to form an aqueous slurry. The slurry was then cast onto the current collector and a uniform coating was formed upon evaporation of water. Activated carbon (Cabot Monarch 1300 pigment black, CMPB, and Kuraray activated carbon powder produced from coconut shell for electric double layer capacitor), SnO_2_-CNT and MnO_2_-CNT electrodes were fabricated by the same cast-evaporation method. A facile tube cell was used to assemble supercapacitors that used a silicone rubber tube containing the electrolyte to join two graphite disc electrodes loaded with the desired active materials^21^.

**Electrochemical measurements** were conducted on a CHI760D electrochemical workstation or a PGSTAT30 Autolab Potentiostat in cells with two or three electrodes. The two electrode cells are the same as the tube cell described above. In all the three electrode measurements, the reference electrode was Ag/AgCl in 3 mol L^−1^ KCl and the counter electrode was a coiled platinum wire or a naked graphite rod. The OCP of an electrode was measured using the CHI760D electrochemical workstation against the Ag/AgCl reference electrode. Prior to the OCP measurement, the electrodes were immersed in the respective electrolytes for up to four hours in order to eliminate the effect of the electrode preparation and obtain more stable OCP values. Simultaneous measurement of OCP and cell voltage was enabled by using a CHI760D electrochemical workstation together with a UNI-T UT60A multimeter. The positive and negative probes of the multimeter were attached to the positive electrode of the supercapacitor and the Ag/AgCl reference electrode respectively. All reported data were based on repeated experiments for at least twice. Errors in potential control and measurement are typically smaller than 1 mV.

## Figures and Tables

**Table 1:** Positive and negative electrode capacitive potential ranges, CPR_+_ and CPR_−_ (vs. Ag/AgCl), and supercapacitor maximum charging voltage, MCV

Supercapacitors configuration	CPR_+_ (V)	CPR_−_ (V)	MCV (V)	Ref
PAN-CNT | HCl | PAN-CNT	0.10 ~ 0.70	0.10 ~ 0.70	0.65	
PPY-CNT | KCl | PPY-CNT	−0.50 ~ 0.50	−0.5 ~ 0.5	1.00	
PEDOT-CNT | KCl | PEDOT-CNT	−0.60 ~ 0.60	−0.6 ~ 0.6	1.20	
MnO_2_-CNT | KCl | MnO_2_-CNT	0 ~ 0.90	0 ~ 0.9	0.90	22
CMPB | K_2_SO_4_ | CMPB	−1.10 ~ 0.80	−1.10 ~ 0.80	1.60	17
CMPB (+) |K_2_SO_4_| CMPB (−) a	−1.10 ~ 0.80	−1.10 ~ 0.80	1.90	17
PPY-CNT (+) | KCl | PEDOT-CNT (−)	−0.50 ~ 0.50	−0.60 ~ 0.60	1.00	
PPY-CNT (+) | KCl | CMPB (−)	−0.50 ~ 0.50	−0.80 ~ 0.60	1.00	
PPY-CNT (+) | KCl | AC (−) b	−0.50 ~ 0.60	−0.90 ~ 0.60	1.50	
PAN-CNT (+) | HCl | CMPB (−)	0.10 ~ 0.70	−0.60 ~ 0.60	1.00	
PAN-CNT (+) | HCl | CMPB (−) a	0.10 ~ 0.70	−0.60 ~ 0.60	1.40	16
PEDOT-CNT (+) | KCl | CMPB (−)	−0.60 ~ 0.60	−0.80 ~ 0.60	1.25	
MnO_2_-CNT (+) | KNO_3_ | AC (−)	0 ~ 0.90	−0.80 ~ 0.60	2.00	5
MnO_2_-CNT(+) | KCl | SnO_2_–CNT (−)	0 ~ 0.90	−0.80 ~ 0.80	1.70	20
MnO_2_-CNT(+) | KNO_3_ | PEDOT (−)	0 ~ 0.90	−0.60 ~ 0.60	1.80	4

^a,^ ^b^ The capacitance ratios of positive to negative electrodes were ^a^ 4:3 and ^b^ 3:1.
